# Structural Transformation of Biochar Black Carbon by C_60_ Superstructure: Environmental Implications

**DOI:** 10.1038/s41598-017-12117-9

**Published:** 2017-09-18

**Authors:** Minori Uchimiya, Joseph J. Pignatello, Jason C. White, Szu-Tung Hu, Paulo J. Ferreira

**Affiliations:** 1 USDA-ARS Southern Regional Research Center, 1100 Robert E. Lee Boulevard, New Orleans, Louisiana 70124 USA; 2 Department of Environmental Sciences, The Connecticut Agricultural Experiment Station, New Haven, Connecticut 06504 USA; 3 Department of Analytical Chemistry, The Connecticut Agricultural Experiment Station, New Haven, Connecticut 06504 USA; 4 Materials Science and Engineering Program, The University of Texas at Austin, Austin, Texas 78712 USA

## Abstract

Pyrogenic carbon is widespread in soil due to wildfires, soot deposition, and intentional amendment of pyrolyzed waste biomass (biochar). Interactions between engineered carbon nanoparticles and natural pyrogenic carbon (char) are unknown. This study first employed transmission electron microscopy (TEM) and X-ray diffraction (XRD) to interpret the superstructure composing aqueous fullerene C_60_ nanoparticles prepared by prolonged stirring of commercial fullerite in water (nC_60_-stir). The nC_60_-stir was a superstructure composed of face-centered cubic (fcc) close-packing of near-spherical C_60_ superatoms. The nC_60_-stir superstructure (≈100 nm) reproducibly disintegrated pecan shell biochar pellets (2 mm) made at 700 °C into a stable and homogeneous aqueous colloidal (<100 nm) suspension. The amorphous carbon structure of biochar was preserved after the disintegration, which only occurred above the weight ratio of 30,000 biochar to nC_60_-stir. Favorable hydrophobic surface interactions between nC_60_-stir and 700 °C biochar likely disrupted van der Waals forces holding together the amorphous carbon units of biochar and C_60_ packing in the nC_60_ superstructure.

## Introduction

Aqueous fullerene C_60_ colloids (nC_60_) prepared by the extended stirring in water or sonication-assisted solvent exchange are frequently considered within the environmental sciences community to be aggregates of individual 60-carbon molecules^1–7^. For example, Chen and Elimelich^8^ described nC_60_ as “alignment of C_60_ molecules”, rather than a new particle or superstructure. Similarly, Murdianti et al^5^. provided a graphical illustration of nC_60_ as the homo-aggregate of individual C_60_ molecules. Choi et al^6^. emphasized that the interaction of C_60_ molecules with water controlled the size of the aggregates composed of 60-carbon molecules. Owning to its low aqueous solubility, which is estimated to be ≈2.63 ng L^−1^ based on toluene extraction^5,9^, C_60_ molecules are postulated to form clusters/aggregates at supersaturation^6^ that further combine to form spherical nC_60_ nanoparticles observable by transmission electron (TEM) and atomic force (AFM) microscopy^1,2,7^. Under this aggregation model, nC_60_ is a stable colloid composed of “randomly arranged, individual C_60_ molecules and their bulk clusters (aggregates)”^1,7^. However, to our knowledge, individual C_60_ molecules have not been visualized in aqueous nC_60_ stock solutions, despite being large enough (≈0.5 nm) to be observed by TEM^10,11^.

An alternative nC_60_ formation mechanism is the physical disintegration of large particles into nanometer-sized particles^12^. Fullerite (commercially available C_60_ crystals) forms particles less than 20 nm when hand-ground^13^ or rubbed between the fingertips^14^. The resulting fullerite nanoparticles have the same face-centered cubic (fcc) crystal plane as the bulk fullerite powder, and form stable aqueous suspensions^14^. The fullerene C_60_ molecule is a near-perfect sphere categorized as a superatom, i.e., a molecule that acts as a stable unit like an atom^15^. Superatomic C_60_ building blocks self-organize to form a regular secondary hierarchical structure, which is called a superlattice, supercrystal, or superstructure^15^. This closely-packed fcc crystal structure of C_60_ bulk powder (fullerite) has long been recognized^16–19^. The present study will refer to the fcc crystal structure of nC_60_ as “superstructure” to describe the secondary hierarchical structure formed by the C_60_ superatoms. The C_60_ “superatoms” in the superstructure are held together by weak van der Waal forces^20,21^.

Previous studies show that the fcc packing of the C_60_ superstructure is preserved in the aqueous nC_60_ formed by sonication of fullerite powder in water (without solvent)^22^, solvent (toluene or THF) exchange^22,23^, prolonged stirring^12^, or grinding and suspension of the ground material^14^. In contrast, highly water soluble fullerol powder (C_60_(OH)_x_, x = 20–24) forms a low-density amorphous aggregate/cluster of varying size (100 to >500 nm)^24^, which is consistent with the aggregation model^1,2^. Because the concept of C_60_ superatoms and their cohesion to form the nC_60_ superstructure is not well recognized within the environmental sciences community^3,5,6^, there is a considerable knowledge gap in the environmental behavior of fullerene, including its solubility, formation, mobility, and surface interactions.

The present study employed TEM and X-ray diffraction (XRD) to first demonstrate the fcc superstructure from nC_60_ suspensions prepared by prolonged (40 d) stirring of fullerite in water. Then, batch retention experiments were conducted to investigate the surface interactions between nC_60_ and chars made from pecan shells. Char is a common constituent of soil originating from historical wildfires, land clearing, crop residue burning, or the intentional application of pyrolyzed biomass wastes (biochar)^25^. Char materials comprise as much as 35% of soil organic carbon^26^. The projected increase in wildfire^27^ and intentional biochar soil amendment^25^ could increase the pyrogenic carbon content of soils. Limited experiments on sludge^28^, soil^3,29,30^, and sand^31^ did not report the nC_60_ mass balance. The present study will explore the following potential interaction mechanisms between char and nC_60_: hydrophobic, van der Waals^20^, and charge repulsion.

## Results and Discussion

### TEM imaging of biochar nanostructures

Biochars are hereby denoted by the feedstock acronym and pyrolysis temperature, e.g., pecan shell feedstock (PS25) and biochar produced at 700 °C (PS700). Grand Canonical Monte Carlo (GCMC) density functional theory (DFT) analysis of CO_2_ isotherm indicated a progressive increase in the surface area of biochars from 271 to 542 m^2^ g^−1^ as a function of pyrolysis temperature (400–700 °C, Table S1). Carbon dioxide measures surface area originating primarily in micropores <1.5 nm in aperture. Because PS700 showed the highest total surface area, TEM was employed to image the nanostructure of PS700.

**Figure 1:**
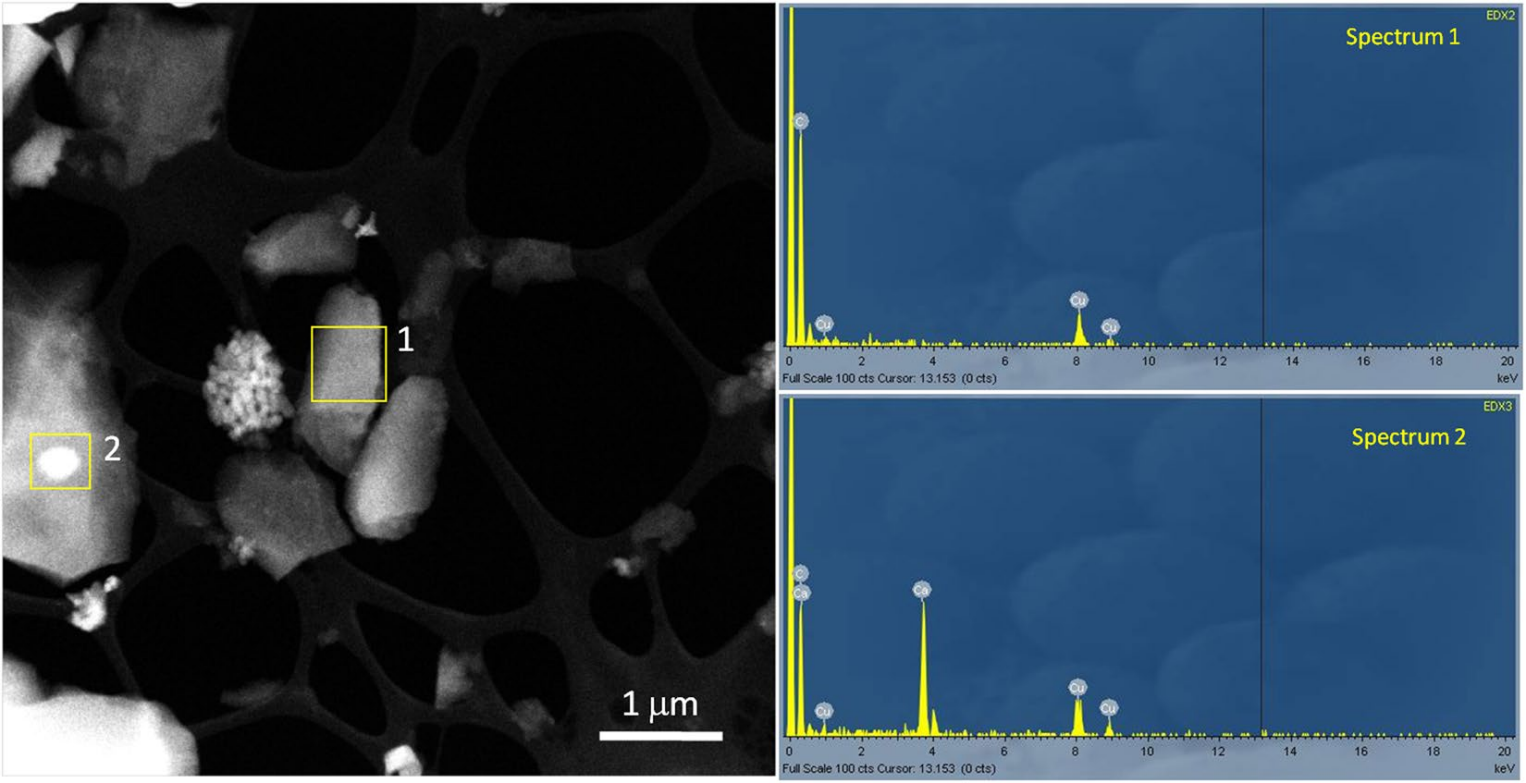
HAADF STEM image and EDS analysis of 700 °C pecan shell biochar. Spectrum 1 acquired from the region within yellow box 1 shows carbon as the only element. Spectrum 2 taken from the bright area within yellow box 2 shows the presence of Ca, C and O. Cu peaks in both spectra are from the TEM grid.

To probe variations in mass/thickness, high angular annular dark field (HAADF) scanning TEM (STEM) was used, as it is sensitive to the atomic number (∝*Z* ^2^)^32^. In this fashion, regions of PS700 having high *Z* are brighter under the HAADF STEM contrast, while regions with low *Z* are darker. Because carbon has a low *Z* value, an accelerating voltage of 120 keV was employed to increase the scattering cross-section and improve image contrast. As shown in Fig. 1, ball-milled PS700 particles of 1–2 µm in size had regions with darker and brighter contrasts. In Fig. 1, yellow squares indicate two locations where EDS signals were obtained. Spectrum 1 (obtained from a particle without brighter contrast) shows carbon as the only element. Spectrum 2 (obtained from a particle with brighter contrast) is dominated by Ca, C, and O signals. Copper peaks in both spectra originate from the Cu grid. Thus, calcium is the cause for the brighter contrast in the STEM-HAADF image. Phase contrast TEM images confirmed that the presence of Ca is related with crystalline CaCO_3_ phase^33^.

**Figure 2:**
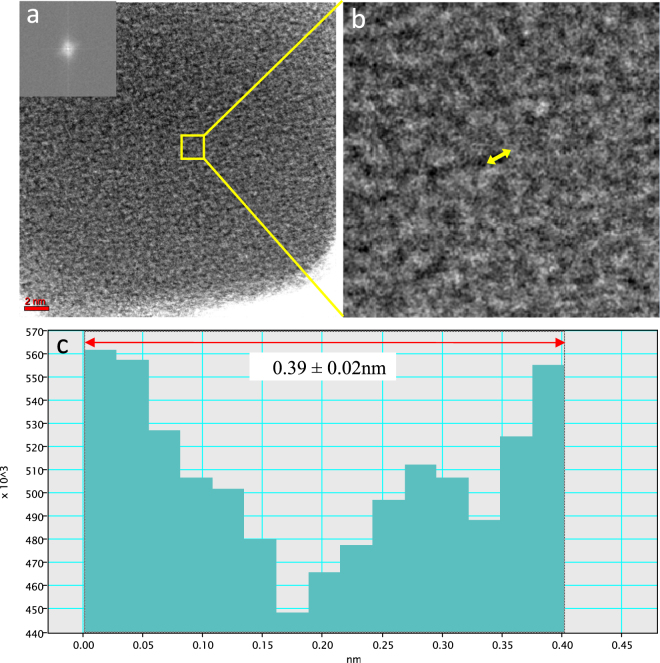
(**a**) Bright-field STEM image of 700 °C pecan shell biochar. The FFT (shown in the inset) confirms that the carbon is amorphous. (**b**) Higher magnification image of a) showing the porosity of the sample (dark spots). (**c**) Intensity profile across the dark spots (yellow arrow) suggests a pore diameter of approximately 0.39 ± 0.02 nm.

The HAADF STEM is a mass-thickness contrast technique, which is sensitive to variations in thickness, and is used to identify meso- (2–50 nm) and macropores (>50 nm)^32^. Fig. 2 shows a bright-field STEM image of PS700. Because the carbon is amorphous (shown by Fast Fourier Transform (FFT) in the inset of Fig. 2a), the contrast primarily results from differences in mass/thickness throughout the amorphous carbon. Pores within the amorphous carbon sample would create a darker contrast in Fig. 2. These dark spots shown in Fig. 2b are likely the locations of micropores measurable by CO_2_ GCMC (Table S1). Based on the intensity profile across these regions (described in detail in Section VIII of Supporting Information), the pore diameter was estimated to be approximately 0.39 ± 0.02 nm (Fig. 2c). For the first time, bright-field STEM was employed to visually estimate the size of pores open to the surface of a black carbon material (amorphous carbon of PS700) to be approximately 0.39 ± 0.02 nm.

### TEM imaging of nC_60_-stir superstructure

**Figure 3:**
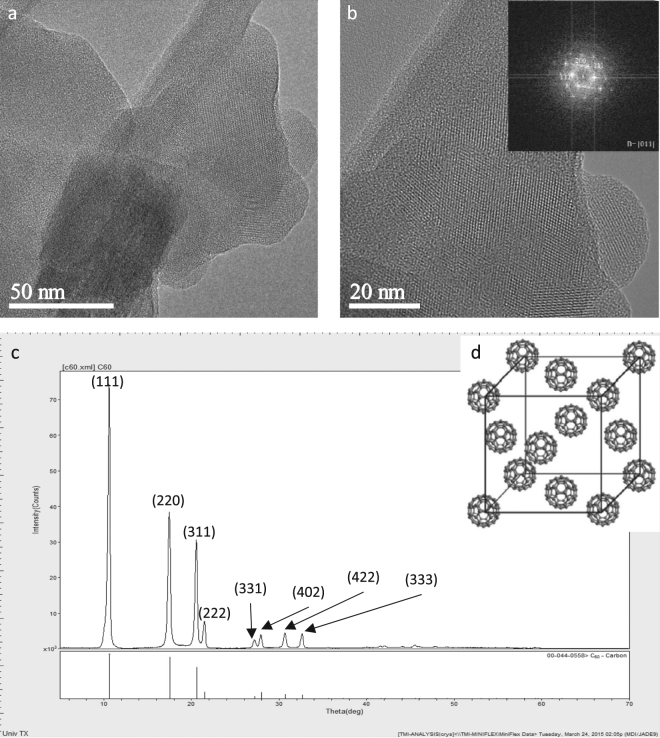
(**a**) Phase contrast TEM images of nC_60_-stir. (**b**) Higher magnification image of a). The FFT shown in the inset indicates a fcc C60 superstructure. (**c**) X-ray diffraction of nC_60_-stir (top spectrum) and C_60_ template from XRD database (bottom spectrum). (**d**) Illustrative fcc C_60_ superstructure^34^.

Bright-field TEM images of nC_60_-stir show lattice fringes, which indicate the presence of crystallinity (Fig. 3a,b); higher magnification images are provided in Figures S4–S5, Supporting Information. The FFT obtained from Fig. 3b (displayed in the inset) reveals that the nC_60_-stir exhibits a face-centered-cubic structure with a lattice parameter, a = 1.356 nm. To better illustrate this configuration, a model^34^ is shown in Fig. 3d. Clearly, the nC_60_-stir is a superstructure formed by C_60_ near-spheres in a fcc configuration with a lattice parameter of 1.356 nm. To further confirm the superstructure of nC_60_-stir, both nC_60_-stir (Fig. 3c, top spectrum) and fullerite (Fig. 3c, bottom spectrum) were analyzed by XRD. The XRD of nC_60_-stir shown in Fig. 3c (top spectrum) matched that of fullerite in the XRD database (bottom spectrum). In conclusion, both TEM and XRD analyses indicate that nC_60_-stir is a superstructure self-assembly of near-spherical C_60_ molecules in a fcc configuration (Fig. 3d), much like the parent fullerite^16^. Although this fcc tendency has been observed in nC_60_-stir^12^, the structural origin has not been explained or interpreted, and was often assumed to be the homo-aggregate of individual C_60_ molecules^1–7^. The polydispersity of nC_60_ superstructure likely controls its dissolution into the toluene extraction fluid used to determine the aqueous “dissolved” C_60_ concentration, [nC_60_]_stock_, as illustrated in the next section.

### Characterization of aqueous nC_60_ stock solutions

**Figure 4:**
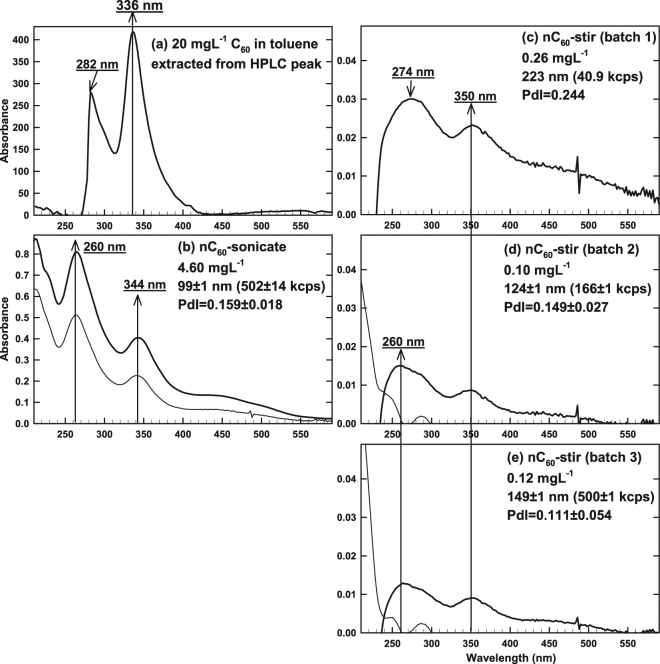
Concentration ([nC_60_]_stock_ determined by UV-HPLC), size, polydispersity index, count rate (from triplicate DLS analyses), and UV/visible spectra (thin lines were obtained immediately after PTFE syringe filtration) of aqueous nC_60_ stock solutions produced by 40 d magnetic stirring (3 batches in **c**–**e**) and sonication (**b**) in this study. C_60_ dissolved in toluene is shown in (**a**) as a reference.

Prolonged stirring of fullerite in water (to prepare nC_60_-stir) is intended to simulate the mixing process^8^ taking place in the environment following an accidental spill of fullerite. In previous studies, as summarized in Section VI of Supporting Information, the reproducibility of nC_60_-stir preparation method had either not been addressed or was called into question^8,35^. Of four separate batches of nC_60_-stir prepared to test the reproducibility of production in the present study, only three produced HPLC-detectable nC_60_-stir (Fig. 4c–e). Although absorbance was low because of the low nC_60_-stir concentration, two broad peaks were observed at 260–274 and 350 nm (Fig. 4c–e), in agreement with the literature;^35,36^ a baseline shift at 800 nm^36^ in some prior reports (which is absent in filtered samples) indicates the presence of unfiltered fullerite. In Fig. 4c–e, nC_60_-stir concentration (by HPLC) and size (by DLS) varied for 3 different batches from 0.10–0.26 mg L^−1^ and 124–223 nm, respectively. As a reference, Fig. 4a shows the UV/visible spectrum of 20 mg L^−1^ C_60_ in toluene corresponding to its HPLC peak.

Because filtration is often used to fractionate nC_60_ into different nm ranges (based on the filter’s pore diameter) before experiments^3,37^, filtered nC_60_ stock solutions were characterized. The thin lines in Fig. 4b (nC_60_-sonicate), and 4c-4e (separate batches of nC_60_-stir) show decreased UV/visible absorbance upon syringe filtration (0.45 µm PTFE). In Fig. 4c–e (nC_60_-stir), the characteristic peaks at 260 and 350 nm disappeared upon filtration. For nC_60_-sonicate (Fig. 4b), the peaks at 260 and 344 nm remained but were at lower intensity after filtration; the concentration of nC_60_-sonicate decreased by an order of magnitude, and resulting low count rate (14.3 kcps at 11 attenuation) did not permit a size measurement by DLS. Other syringe filters (0.45 µm cellulose acetate and nylon, VWR) lead to similar reduction in the absorbance of nC_60_-sonicate, even though all stock solutions in Fig. 4 had been previously vacuum-filtered through 0.45 µm cellulose acetate membrane filter, as described in the Methods section. Based on above-described influence of syringe filtration, only toluene layer (and not aqueous layer) was filtered prior to the HPLC quantification of C_60_ in the subsequent sections. Above observations suggest that intended size fractionation of nC_60_ via filtration, often employed in the prior reports^3,37^, could filter out the particles smaller than the filter’s pore diameter.

### Surface interactions between nC_60_ supercrystals and 2-mm biochar pellets

**Figure 5:**
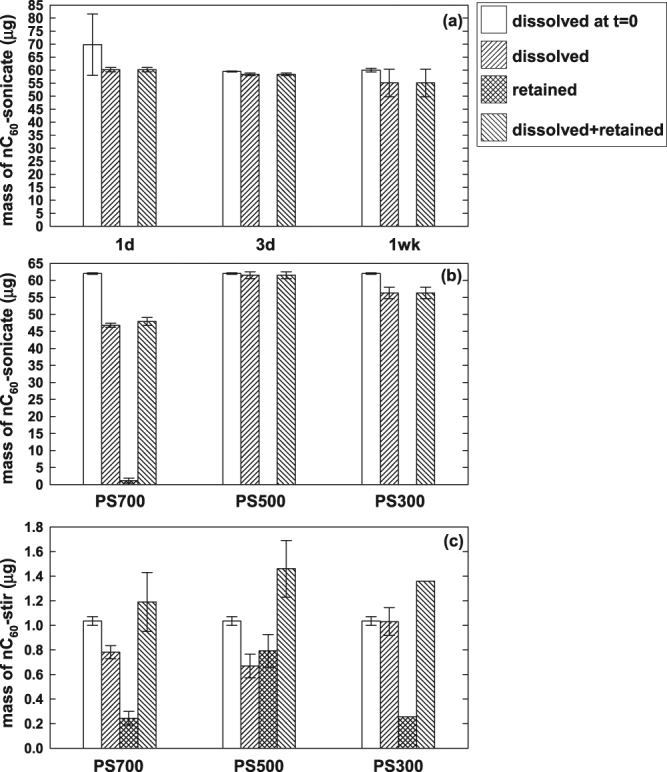
Mass fraction of nC_60_ (in μg) that was dissolved at t = 0 (calculated based on [nC_60_]_stock_ of duplicate controls at each sampling time), dissolved at the sampling time (based on [nC_60_]_dissolved_), retained on biochar (based on [nC_60_]_retained_), and the mass balance (dissolved+retained) from retention kinetics of nC_60_-sonicate on PS300 (**a**) and 1 d equilibration of nC_60_-sonicate (**b**) and nC_60_-stir (**c**) on PS300-700 at 5 (**a**,**c**) and 20 g L^−1^ (**b**) biochar loadings. All values are given as mean ± s.d. of duplicate experiments.

Figure 5 presents the mass distribution (in μg) of nC_60_-sonicate (a-b) and nC_60_-stir (c) in mean ± s.d. from duplicate retention experiments. In Fig. 5, nC_60_ mass fractions were determined based on [nC_60_]_dissolved_ for “dissolved” portion of nC_60_ in water at the sampling time, and [nC_60_]_retained_ for biochar-associated “retained” fraction, as described in detail in Methods section. Mass balance was determined as the sum of dissolved and retained fractions. The “dissolved at t = 0” fraction was based on [nC_60_]_stock_ measured at each sampling time using the control reactors containing nC_60_-stir or nC_60_-sonicate alone, without biochar. Figure 5 in the unit concentration and [nC_60_]_retained,calc_ (Equation 1) are provided in Figure S6, Supporting Information.

Kinetic experiments were first performed at 5 g L^−1^ PS300 using nC_60_-sonicate that is an order of magnitude more concentrated than nC_60_-stir (Fig. 4). As shown in Fig. 5a, dissolved mass of nC_60_-sonicate remained constant over 1 wk period in the presence of low pyrolysis temperature (300 °C) biochar. Hot toluene extraction of oven-dried biochar after the retention experiment did not recover nC_60_-sonicate during the timecourse of the experiment (Fig. 5a). As a result, the C_60_ mass balance (dissolved+retained) equaled the dissolved fraction over 1 wk period.

To investigate the influence of pyrolysis temperature and biochar loading, 1-day equilibration experiment was conducted at a higher loading (20 g L^−1^) of 300, 500, and 700 °C biochars (Fig. 5b). Higher biochar loading did not significantly decrease the dissolved nC_60_-sonicate fraction in the presence of PS300 (5–20 g L^−1^ in Fig. 5a,b). Because of negligibly low solid-associated “retained” fraction, the mass balance of nC_60_-sonicate was within the error range of the dissolved fraction (PS700 < PS300 < PS500). In conclusion, the retention of nC_60_-sonicate by 5–20 g L^−1^ of 300–700 °C biochars was negligibly low. The results in Fig. 5a,b suggest charge repulsion between hydroxyl-enriched nC_60_-sonicate and carboxyl-enriched PS300; both are negatively charged^8,38^. Retained fraction of nC_60_-sonicate was observed only on PS700 (Fig. 5b), which relative to PS300 is more hydrophobic and contains fewer oxygen-containing functional groups^38,39^.

Figure 5c presents the retention of nC_60_-stir on 5 g L^−1^ of 300, 500, and 700 °C biochars. At an order of magnitude lower concentration of nC_60_-stir than nC_60_-sonicate, a significant fraction of nC_60_-stir was retained by biochars in the order, PS300 ≈ PS700 < PS500 (“retained” in Fig. 5c). Dissolved nC_60_-stir fraction followed the order, PS500 ≈ PS700 < PS300. The mass balance did not show a clear temperature trend, and exceeded the dissolved fraction at t = 0 for PS500 having the highest retained fraction. This could originate from the greater extraction efficiency of nC_60_-stir from dried biochar (using hot toluene method) than from water. In conclusion, the recovery of nC_60_-stir retained by the biochar was greater than nC_60_-sonicate, despite an order of magnitude lower [nC_60_]_stock_ of nC_60_-stir than nC_60_-sonicate. The nC_60_-sonicate is likely to contain higher amounts of hydroxyl substituent than nC_60_-stir, because of the sonication process^22^ incurring radical formation^40^. Favorable hydrophobic interactions between nC_60_-stir and biochars likely drove the formation of biochar-associated nC_60_-stir in Fig. 5c.

**Figure 6:**
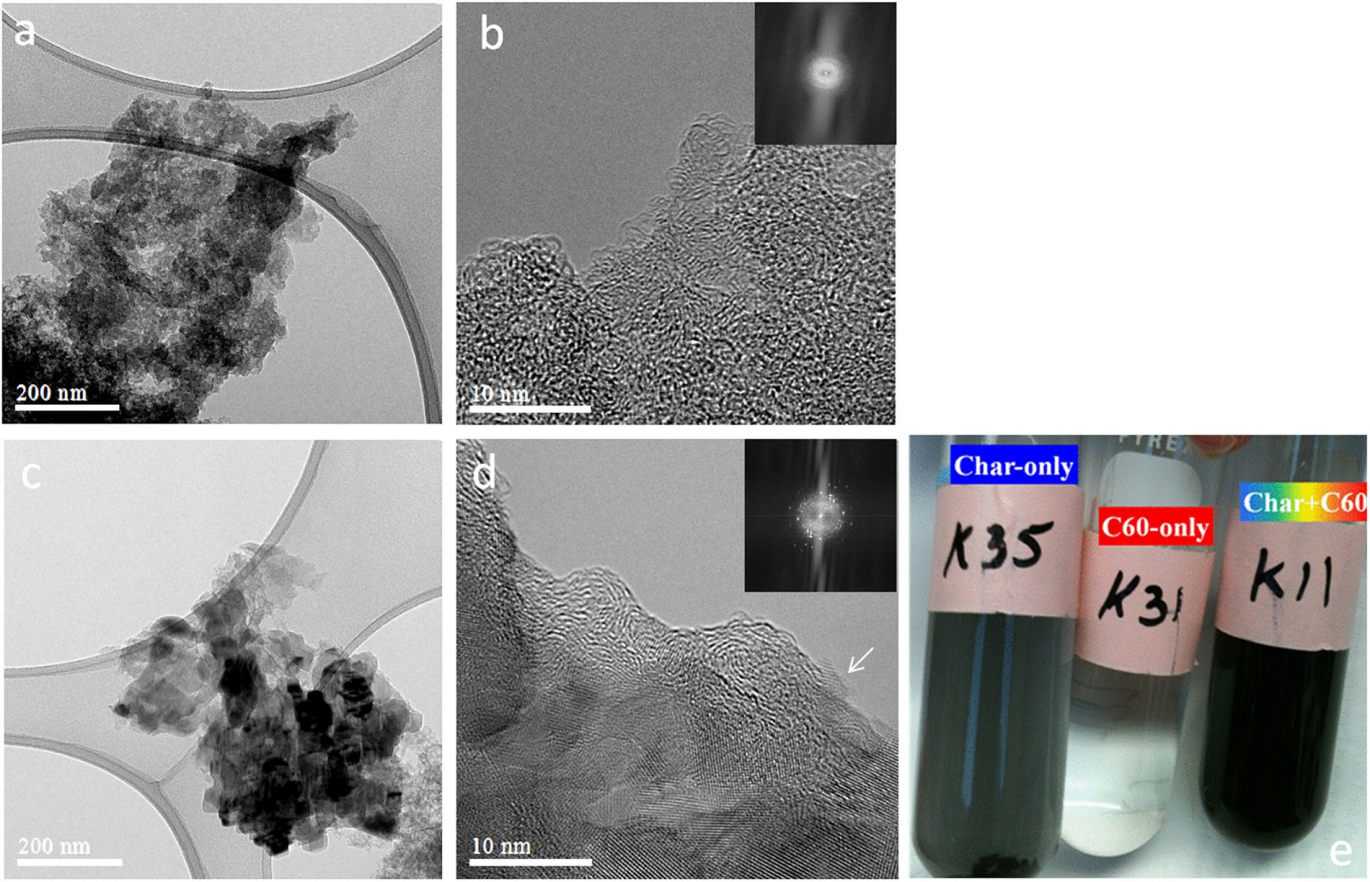
TEM images of PS700+nC_60_-stir. (**a**) Amorphous structure. (**b**) Higher magnification of (**a**). (**c**) Crystalline structure. (**d**) Higher magnification of (**c**). (**e**) Disintegration of 2-mm PS700 pellets by nC_60_-stir after 3 d equilibration (initial conditions: 0.15 g PS700, 0.149 mg L^−1^ nC_60_-stir, and 10 mM NaCl). The insets in (**b**) and (**d**) confirm the amorphous and crystalline nature of structures.

When high temperature (700 °C) biochar was equilibrated with nC_60_-stir at a sufficiently high biochar loading (≥20 g biochar L^−1^), 2-mm biochar pellets reproducibly disintegrated to form a homogeneous, stable, black-colored aqueous colloidal suspension (Fig. 6e). The PS700+nC_60_-stir suspension (Fig. 6e, far right) was produced at >30,000 biochar/nC_60_ ratio by weight, and contained measurable [nC_60_]_dissolved_. The suspension was stable after the supernatant containing nC_60_-stir was replaced by water (Figure S7 top far right, Supporting Information). The disintegration of biochar pellets was not observed at lower biochar loadings (Figure S7 top), when low pyrolysis temperature biochar was employed (PS350 in Figure S7, bottom), or when nC_60_-sonicate was employed instead of nC_60_-stir. Higher temperature biochars have higher attrition^41^ to form smaller particles by mechanical forces^42,43^. Higher biochar loading could enhance the mechanical crushing of biochar pellets during the end-over-end rotation in the presence of nC_60_-stir. Hydrophobic interactions between PS700 and nC_60_-stir (but not nC_60_-sonicate) lead to the retention of nC_60_-stir (Fig. 5c) accompanied by the disintegration of biochar (Fig. 6). Collectively, hydrophobic interactions drove the retention of nC_60_-stir (but not nC_60_-sonicate having higher aqueous solubility suggesting greater hydrophilicity, Fig. 4) on biochar, and induced structural transformation of amorphous carbon (PS700) in the presence of crystalline carbon (nC_60_-stir).

Figure 6a–d show bright-field TEM images of PS700+nC_60_-stir suspension (6e, far right). Figure 6a,b indicate the formation of amorphous carbon (FFT shown in inset of Fig. 6b) nanoparticles from PS700 pellets, in the presence of nC_60_-stir. Spaghetti-like amorphous carbon is composed of random carbon domains with defects linked by the aliphatic carbon chains^44^. On the other hand, Fig. 6c,d show a polycrystalline structure composed by nanocrystals having different orientations, as confirmed by multiple diffraction spots (FFT inset of Fig. 6d). However, the lattice spacing was not consistent with the C_60_ superstructure (Fig. 3), and thus could originate from CaCO_3_ in pecan shell biochar (Fig. 1)^41^. It is inherently challenging to distinguish two carbon materials (char and C_60_) by TEM because of low contrast and overlapping projection^30,45^. However, Fig. 6d shows graphitic structures (arrow) on the edge of amorphous carbon that could originate from the decomposition of nC_60_-stir superstructure to form C_60_ molecules. This graphitic structure was not observed in biochar alone (Figs 1, 2) or nC_60_-stir alone (Fig. 3).

**Figure 7:**
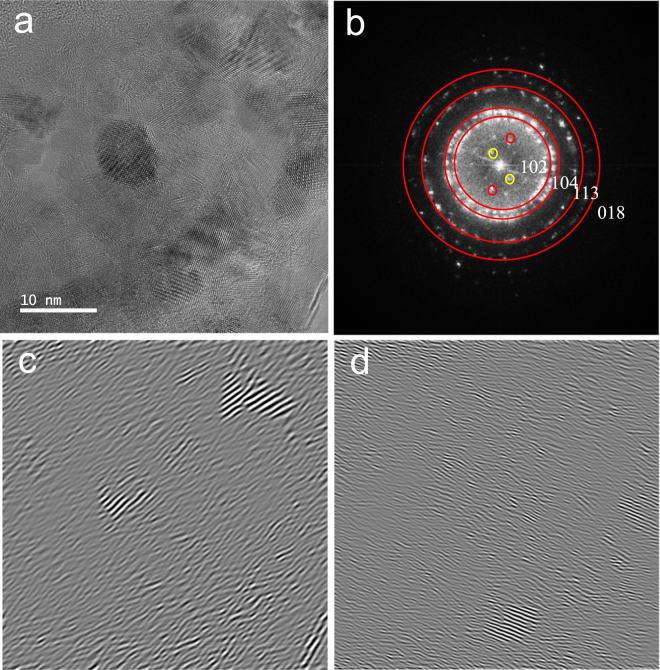
(**a**) Phase contrast TEM image of C60 superstructure in nC60+PS700. (**b**) FFT of image (**a**) showing the presence of CaCO_3_ represented by the diffraction rings and the C60 superstructure defined by the yellow and red diffraction spots. (**c**) Inverse FFT produced by the (200) yellow diffraction spots of the C60 superstructure. (**d**) Inverse FFT produced by the (220) red diffraction spots of the C60 superstructure.

Figure 7a focuses on the polycrystalline structure within the phase contrast TEM image of PS700+nC_60_-stir. Nanocrystals having different orientations are confirmed by the multiple diffraction spots and lattice fringes in different directions (Fig. 7b is the FFT image of Fig. 7a). By indexing the FFT image, all diffraction rings matched CaCO_3_ planes: (102), (104), (113), and (018) towards outer rings in Fig. 7b. However, both yellow and red diffraction spots in Fig. 7b matched the lattice spacing of C_60_ superstructure: 0.49 nm of (220) plane (red spots in Fig. 7b), and 0.795 nm of (200) plane (yellow spots). In Figures 7c (for yellow spots in Figure 7b) and 7d (for red spots in Figure 7b), an inverse FFT technique was employed to visualize the retained fraction of nC_60_-stir in PS700. As shown in Fig. 7, the inverse FFT on the red and yellow diffraction spots revealed the C_60_ superstructures embedded within PS700, i.e., [nC_60_]_retained_.

In conclusion, Figs 4–7 re-emphasize that nC_60_ is a superstructure, rather than the homo-aggregate of C_60_ molecules. If nC_60_ was the aggregate of individual C_60_ molecules, penetration into microporous networks is expected. Such reaction is expected to be highly irreversible, and will be controlled by the abundance of micropores (<2 nm)^46,47^, which progressively increases from 400 to 700 °C (Table S1). In contrast, van der Waals and hydrophobic interactions involving the polyaromatic surface of PS700 will favor the heteroaggregation of nC_60_ superstructure. Biochar’s hydrophobicity progressively increases as a function of pyrolysis temperature, resulting in the lower H/C atomic ratio (Table S1). Heteroaggregation of nC_60_-stir with PS700 (Fig. 5c) and associated hydrophobic interactions could disrupt the relatively weak van der Waals forces holding together (i) amorphous carbon units^44^ of biochar and (ii) C_60_ packing of nC_60_ superstructure^20,21,48^.

Physical disintegration of biochar particle by the engineered carbon nanomaterial (nC_60_, Fig. 6) without sonication^49^, will pose a number of environmental consequences. Environmental transport of pyrogenic carbon is strongly size-dependent^50^, and constitutes a significant proportion of the global carbon cycle^51^. The presence of hydrophobic carbon nanoparticles, like nC_60_-stir occurring from accidental spill, will promote the transport of biochar soil amendment^52^ by producing biochar nanoparticles (Fig. 6) and composites (Fig. 7). Produced biochar nanoparticles will have additional environmental consequences^53^, including the off-site migration of sorbed pollutants^25^.

## Methods

Distilled, deionized water (DDW) with a resistivity of 18 MΩ cm (APS Water Services, Van Nuys, CA) was used in all procedures. Unless otherwise noted, all chemical reagents were obtained from Sigma-Aldrich (Milwaukee, WI) at the highest purity available.

### Pecan shell biochars

As described in detail previously^49,54^, pecan shell feedstock (PS25) was ground (SM 2000 cutting mill, Retsch Gmbh, Haan, Germany) and sieved (<2 mm) prior to pyrolysis at 300, 350, 400, 500, 600, or 700 °C under a flow rate of 1,600 mL min^−1^ N_2_ for 4 h using a laboratory scale box furnace (22 L void volume) with a retort (Lindberg, Type 51662-HR, Watertown, WI). Biochar products were allowed to cool to room temperature overnight under the N_2_ atmosphere. Proximate and ultimate analysis results^54^ and N_2_ and CO_2_ isotherms-based surface area and porosity are summarized in Table S1 of Supporting Information.

### Aqueous nC_60_ stock solutions

Two published methods were used. In the first^8,55,56^, bulk fullerene C_60_ powder (99.9% purity fullerite; Materials and Electrochemical Research, Tucson, AZ) was magnetically stirred in DDW (1.0 g L^−1^) in the dark for 40 d. The suspension was initially black and gradually turned brown over the 40 d stirring period. The suspended particles were removed by the vacuum filtration (DDW pre-rinsed 0.2 μm cellulose acetate membrane; Sartorius, Bohemia, NY) to produce a clear stock solution having a light yellow/brown hue and a pH of 6.0 (Sartorius Professional meter PP-15). The resulting stock solution is denoted nC_60_-stir. The second method^57^ employed a sonication probe (450 Sonifier, Branson Ultrasonics, Danbury, CT) to increase the nC_60_ concentration by the oxidative formation of hydroxyl substituents^22,37^. Five mL of C_60_ powder dissolved in toluene (1.2 g L^−1^ clear purple solution) was added to a solution composed of 50 mL DDW and 1.5 mL ethanol. Figure S1 of Supporting Information shows the resulting solution having toluene (top) and aqueous (bottom) layers^58^. This solution was sonicated by directly inserting the probe for 3 h while periodically adding DDW to replace the water evaporated as a result of the exothermic sonication process. Sonication caused the solution to develop a cloudy yellow-white color (Figure S2), and the final solution (after toluene was driven off by the heating) was clear yellow. This solution was vacuum filtered through 0.45 μm (cellulose acetate) and then 0.2 μm (cellulose nitrate) membrane filters. The resulting stock solution is denoted nC_60_-sonicate (Figure S3). An analogous procedure was followed to produce nC_70_ stock solution by sonication (nC_70_-sonicate characterized in the Section VII of Supporting Information) to use as the internal standard in HPLC quantification of nC_60_. All aqueous fullerene stock solutions (nC_60_-stir, nC_60_-sonicate, and nC_70_-sonicate) were stored at 25 °C in the dark, and were stable for several months, as reported in the literature^59^. The stock solutions were characterized by UV/visible spectrophotometry (HP8452A, Hewlett-Packard, Palo Alto, CA) with DDW as the blank. Hydrodynamic diameter was determined by dynamic light scattering (DLS; Zetasizer NanoZS, Malvern, Westborough, MA). All DLS analyses were performed in triplicate using the disposable sizing cuvette at the material RI of 2.20, attenuation of 11, water as the dispersant, and by the default general method algorithm; count rate (in kcps) and polydispersity index (PdI) were recorded in addition to the hydrodynamic diameter.

### Surface interaction of nC_60_ with biochars

Batch experiments were conducted in duplicate using amber glass vials with Teflon-lined screw caps (40 mL nominal volume, Thermo Fisher Scientific, Waltham, MA) at 5–20 g biochar L^−1^; 30 mL of undiluted nC_60_ stock solution was added directly to dry 2-mm biochar pellets. Reactors were equilibrated by shaking end-over-end at 70 rpm. Control experiments were conducted for the nC_60_ stock solution without biochar, and biochar without nC_60_ stock solution, each in duplicate. At each sampling time, biochar was allowed to settle for 1 h, and then the supernatant was carefully decanted into a new glass vial. The supernatant was mixed with 200 g L^−1^ NaCl stock solution to yield 1 wt% NaCl. The NaCl was used to facilitate the transfer of nC_60_ from the aqueous to toluene phase, and to prevent emulsion^9^. After vigorous shaking by hand, 4 mL toluene (HPLC grade) was added, and the reactor was vortexed and then rotated at 70 rpm overnight. After allowing the two (water and toluene) layers to separate, only the toluene layer was syringe filtered (0.45 μm Millipore Millex-GS; Millipore, Billerica, MA), and 200 µL filtrate was injected into HPLC system with diode array detector (Agilent Technologies, Santa Clara, CA) and Cosmosil Buckyprep-M Packed column (4.6 × 2500 mm; SES Research, Houston, TX). The HPLC column was designed to separate C_60_ from C_70_ in pure toluene mobile phase at 1.0 mL min^−1^ flow rate, and C_60_ was quantified at λ_max_ of 336 nm in toluene (Fig. 4a) and the retention time of 8 min^60^. Determined nC_60_ concentration is hereby denoted [nC_60_]_dissolved_. The same procedure was used to determine C_60_ concentrations in nC_60_-stir and nC_60_-sonicate stock solutions hereby termed [nC_60_]_stock_.

The portion of nC_60_ retained by biochar (hereby termed [nC_60_]_retained_) was independently quantified by a hot toluene extraction method^61^. Biochar remaining in each reactor (after decanting supernatant; residual supernatant was determined gravimetrically) was transferred to a clean vial using DDW, and then oven-dried at 45 °C overnight. After recording the weight of oven-dried biochar in the new vial, 2 mL toluene was added, and the resulting biochar suspension in toluene was immersed in 65 °C water bath for 6 h. The reactor was vortexed and rotated at 70 rpm overnight, and then syringe filtered (0.45 μm) for the HPLC analysis of C_60_, as described above. Solid-associated nC_60_ was also calculated as [nC_60_]_retained,calc_ (in mg g^−1^) using the equation below, and compared with [nC_60_]_retained_:^62^

1 $${[{{\rm{nC}}}_{{\rm{60}}}]}_{\mathrm{retained},\mathrm{calc}}=\frac{{{\rm{V}}}_{{\rm{s}}}}{{\rm{m}}}({[{{\rm{nC}}}_{{\rm{60}}}]}_{{\rm{stock}}}-{[{{\rm{nC}}}_{{\rm{60}}}]}_{{\rm{dissolved}}})$$

where V_s_ is the total volume (30 mL), and m (in g) is the dry weight of 2-mm biochar pellets.

To determine the portion of nC_60_ retained by the reactor (hereby termed [nC_60_]_vial_), both the vial and cap of the amber glass reactor were washed thoroughly 3 times with DDW to remove residual supernatant containing nC_60_. Washed reactors were air dried, and then 2 mL toluene was added. The capped reactor was then vortexed and rotated at 70 rpm overnight, and then syringe filtered (0.45 μm) for the HPLC analysis. The resulting [nC_60_]_vial_ was determined to be negligible for all experiments presented in this study.

In each experiment, mass balance (in μg C_60_) was calculated based on [nC_60_]_dissolved_ (solution-phase nC_60_ concentration at the sampling time) and [nC_60_]_retained_. The mass balance was compared with μg of nC_60_ added to each reactor at t = 0, which was calculated based on the reactor volume (30 mL) and [nC_60_]_stock_ determined at each sampling time using the controls containing nC_60_ stock solution without biochar.

### TEM imaging of nC_60_-stir and biochar before and after the reaction

Ball-milled and sieved (400 mesh, <37 µm) PS700 was sonicated in ethanol for 15 min. One drop of the resulting suspension was deposited on a 200 mesh carbon-lacey Cu grid. To prepare samples containing nC_60_-stir, two drops of nC_60_-stir before and after the reaction with PS700 were deposited on the grids. TEM images were obtained using a JEOL 2010F TEM (JEOL USA, Peabody, MA) operated at 120 kV, coupled with an energy-dispersive x-ray spectroscopy (EDS). As widely described in the literature, TEM observations could modify the structure of aqueous colloids if exposed to high acceleration voltages^10^, drying or addition of surfactants/solvents during sample preparation^63^, or by freezing employed during the cryogenic TEM. With this in mind, the present study employed 120 kV and short exposure times to minimize these experimental artifacts.

### X-ray diffraction

The crystalline structures of nC60-stir stock solution and the bulk fullerite powder were characterized by X-ray diffraction (XRD) with a Philips X’pert diffractometer (with Cu Kα radiation) using a step-scan mode in the range of 10° to 110° with intervals of 0.03° and wavelength of 1.5406 Å (Cu Kα). XRD computer simulations were carried out using a Diamond 3.2e2 software.

## Electronic supplementary material

Supplementary Information
