# Supplementary material for: Structural Transformation of Biochar Black Carbon by C60 Superstructure: Environmental Implications
Source: Sci Rep. 2017 Sep 18;7:11787. doi: 10.1038/s41598-017-12117-9 (PMC5603586; doi:10.1038/s41598-017-12117-9)
Supplement: Supplementary file 1 — Supplementary Information [file 41598_2017_12117_MOESM1_ESM.pdf]

Supporting Information for

Structural Transformation of Biochar Black Carbon by C_{60} Superstructure: Environmental Implications

Minori Uchimiya[*,a]*, Joseph J. Pignatello*[b]*, Jason C. White*[c]*, Szu-Lung Hu*[d]*, Paulo J. Ferreira*[d]

[a]*USDA-ARS Southern Regional Research Center, 1100 Robert E. Lee Boulevard, New Orleans, Louisiana 70124, USA*

[b]*Department of Environmental Sciences, The Connecticut Agricultural Experiment Station, New Haven, Connecticut 06504, USA*

[c]*Department of Analytical Chemistry, The Connecticut Agricultural Experiment Station, New Haven, Connecticut 06504, USA*

[d]*Materials Science and Engineering Program, The University of Texas at Austin, Austin, Texas 78712, USA*

*Corresponding author fax: (504) 286-4367, phone: (504) 286-4356, email: sophie.uchimiya@ars.usda.gov

Number of pages: 12

Number of tables: 2

Number of figures: 8

I. Biochar characterization procedures and results

Proximate analysis. Moisture, ash, volatile matter (VM), and fixed carbon contents of biochars were determined in triplicate by following American Society for Testing and Materials (ASTM) method D7582[1] using LECO thermogravimetric analyzer (TGA701, LECO, St. Joseph, MI). Moisture was determined as the weight loss after heating the sample under N_2 atmosphere in an open crucible to 107 °C and holding at this temperature until sample weight stabilized. The VM was determined as the weight loss after heating the sample under N_2 atmosphere in a covered crucible to 950 °C and held for 7 min. Ash was defined as the remaining mass after subsequently heating the sample under O_2 atmosphere in an open crucible to 750 °C and holding at this temperature until sample weight stabilized. After the determination of moisture, ash, and VM, fixed carbon was calculated by difference.

Ultimate analysis. Elemental composition (CHNSO) was determined by dry combustion using Perkin-Elmer 2400 Series II CHNS/O analyzer (Perkin-Elmer, Shelton, CT).

Boehm titration. Total acidity was estimated using a modified Boehm titration method.[2] Briefly, 10 g L^{-1} char suspension was prepared in 0.1 M NaOH. After stirring for 24 h, char suspension was filtered (0.45 μm Millipore Millex-GS; Millipore Corp., Billerica, MA) and 9 mL of 0.1 M HCl was added to 4.5 mL filtrate. Resulting solution was N_2 sparged for 2 h and then titrated with 0.1 M NaOH (titrando 835 autotitrator, Metrohm ion analysis, Herisau, Switzerland). Blanks were prepared by adding 4.5 mL of 0.1 M NaOH to 9 mL of 0.1 M HCl. Surface acidity (in mequiv g^{-1}) was determined assuming that NaOH neutralizes all organic acids with pK_a less than 12, including high pK_a phenols.[2]

Biochar characterization results. Ultimate (C, H, N, S, and O; weight-percent on a moisture- and ash-free basis) and proximate (ash, fixed C, moisture, and volatile matter in weight-percent

on a moisture-free basis) analysis results for 300-700 °C pecan shell biochars are presented in Table S1. Table S1 also presents atomic H/C ratio as an index of aromaticity, and O/C as an index of polarity and hydrophilicity. The trends with temperature in Table 1 are in agreement with the literature.[3,4] Grand Canonical Monte Carlo Density Functional theory (GCMC) analysis of CO_2 isotherm indicated a progressive increase in surface area of 271-542 m^2 g^{-1} from 400 to 700 °C (Table 1). Low N_2 BET surface area of biochars has been widely reported,[5] and is attributed to the diffusion limitation under the liquid N_2 temperature.[6] Point of zero charge (PZC by electrophoretic mobility) of biochars is typically below 3, regardless of pyrolysis temperature.[7] Therefore, both biochar[7] and nC_{60}[8] are expected to be negatively charged within the pH range employed in the present study.

Table S1. Ultimate[a] and proximate[b] analysis results of pecan shell biochars pyrolyzed at 300-700 °C.[9] Values are given as mean±s.d. of triplicate analysis. Surface area and porosity were determined by N_2[c] and CO_2[d] isotherms.

	ultimate analysis							proximate analysis				surface area		porosity
biochar	C	H	N	S	O	atomic ratio		ash	fixed C	Moisture	VM	m^2 g^{-1}		cm^3 g^{-1}
	wt%					H/C	O/C	wt%				N_2 BET	CO_2 GCMC (cumulative)	
PS300	68±4	6.6±0.3	0.57±0.04	1.4±0.1	24±4	1.16	0.26	1.94±0.03	41.9±0.5	2.1±0.1	56.2±0.5	2.58		
PS350	75±1	5.3±0.3	0.64±0.04	0.75±0.05	19±1	0.84	0.19	2.78±0.03	59.9±0.1	2.69±0.04	37.3±0.1	1.50		
PS400	79±1	5.1±0.2	0.65±0.04	0.5±0.2	15±1	0.76	0.14	3.13±0.02	65.6±0.1	2.9±0.1	31.3±0.1	1.10	271	0.08
PS500	89±1	3.8±0.1	0.7±0.1	0.46±0.01	6±1	0.51	0.05	3.8±0.1	76.7±0.1	2.4±0.1	19.5±0.1	0.95	387	0.11
PS600	92.8±0.4	3.2±0.1	0.81±0.03	0.5±0.1	2.7±0.5	0.41	0.02	4.1±0.1	83.7±0.3	2.33±0.01	12.2±0.3		479	0.13
PS700	95.1±0.3	1.7±0.5	0.7±0.3	0.1±0.1	2±1	0.21	0.02	4.47±0.05	86.9±0.1	2.45±0.02	8.7±0.2		542	0.15

[a]Corrected to moisture- and ash-free. [b]Corrected to moisture-free. [c]Based on Brunauer-Emmett-Teller fit of the N_2 adsorption isotherm at 77 K. [d]Based on Grand Canonical Monte Carlo Density Functional theory analysis of the CO_2 isotherm at 273.15 K.

II. Preparation of nC_{60}-sonicate

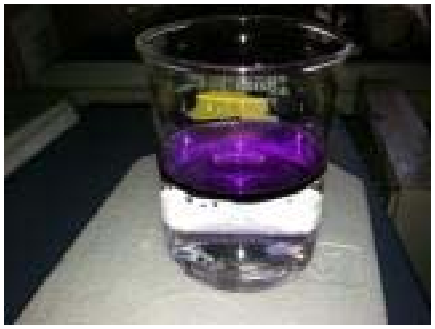

Figure S1. Toluene (upper purple) and aqueous (lower transparent) layers are visible when fullerite powder dissolved in toluene is added to to water+ethanol.

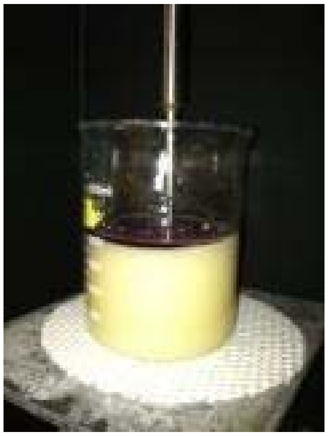

Figure S2. Color change during sonication.

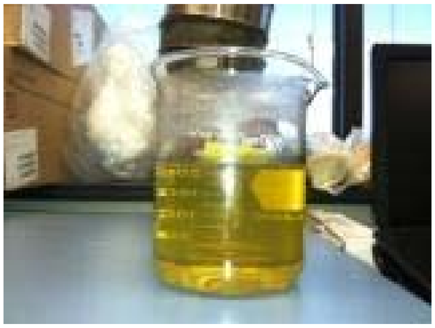

Figure S3. Final nC_{60}-sonicate stock solution.

III. Bright-field TEM images of nC_{60}-stir at higher magnification

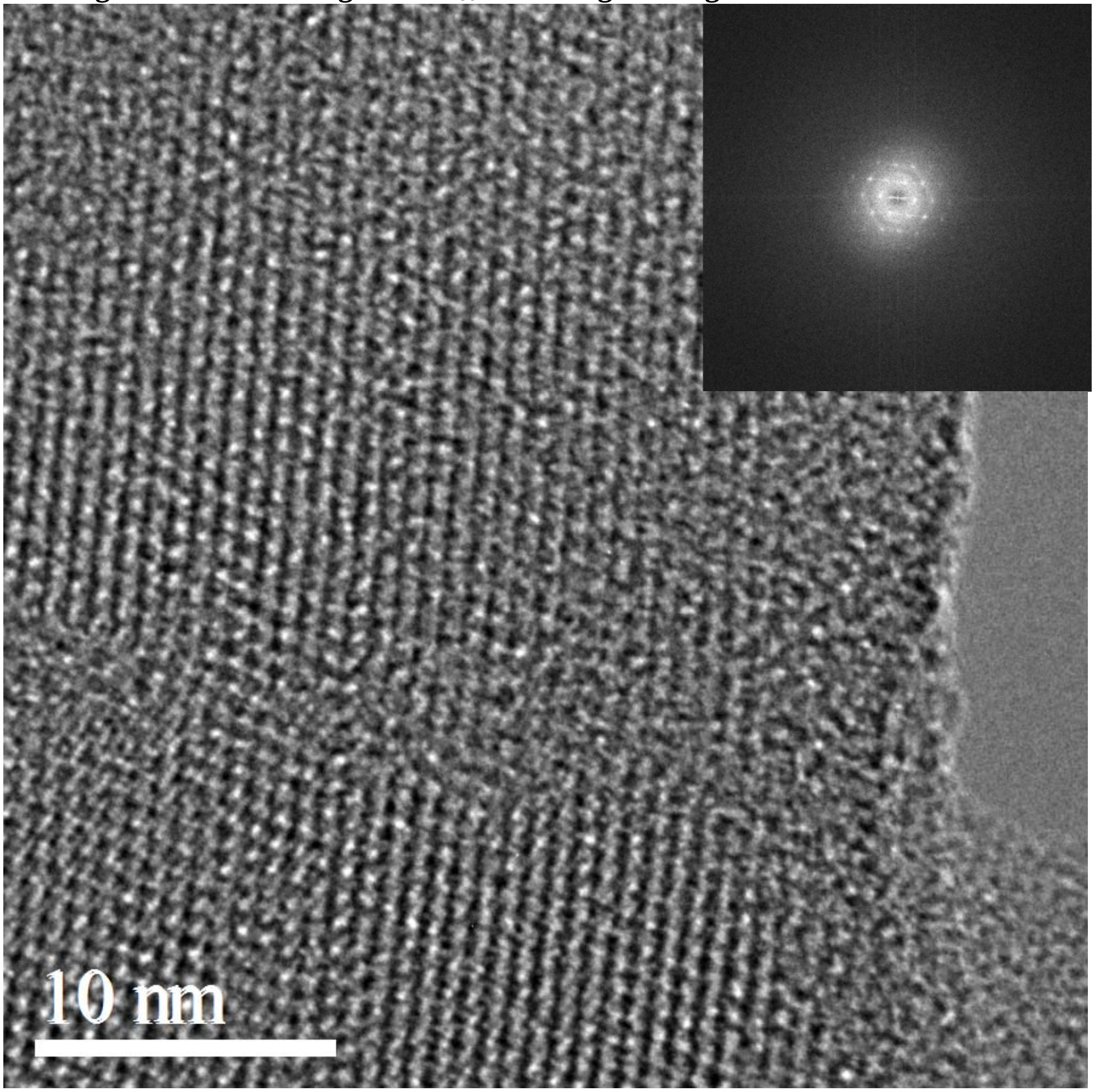

Figure S4. Bright-field TEM image of at higher magnification (300 k).

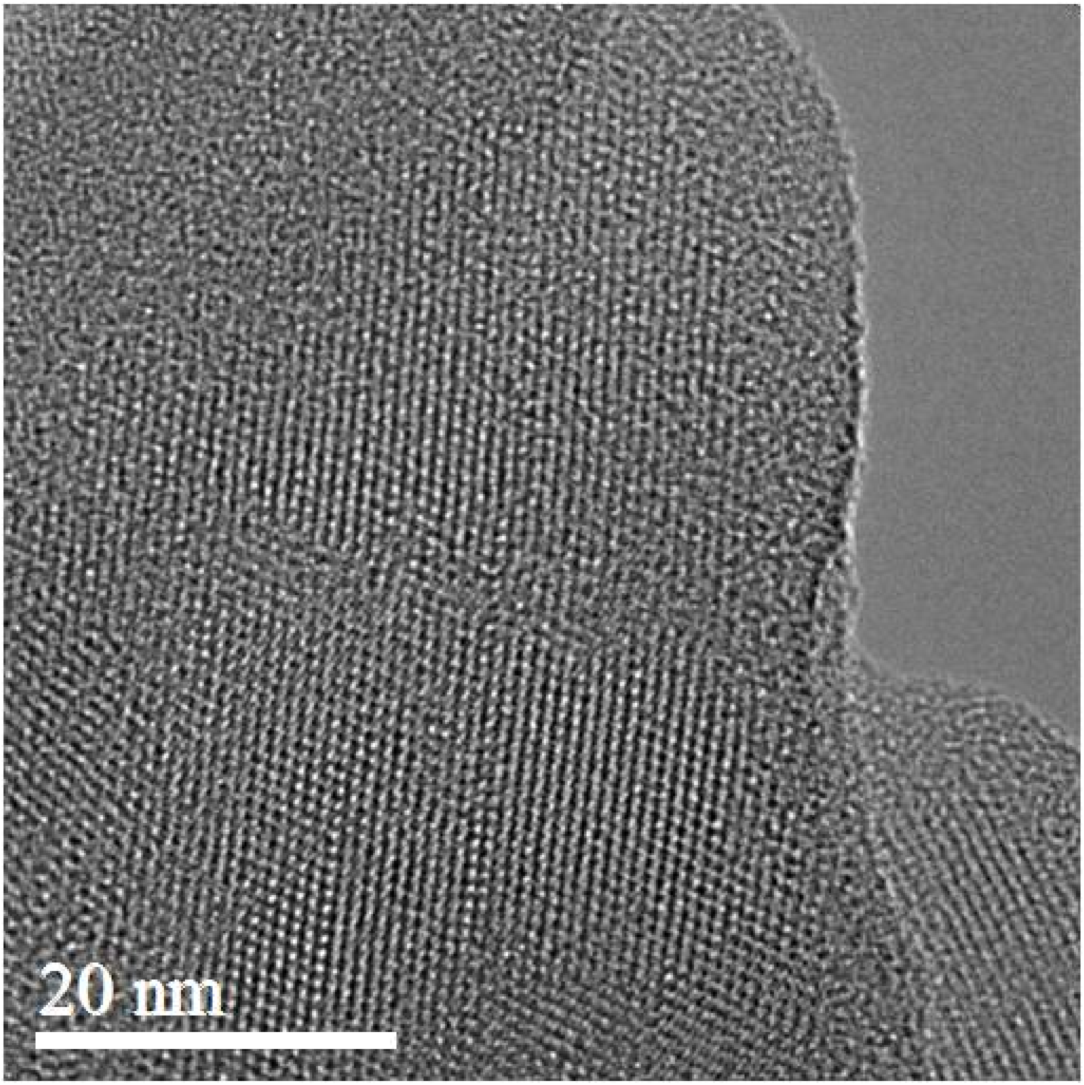

Figure S5. Bright-field TEM image of at higher magnification (300 k).

IV. Figure 5 in the unit concentration

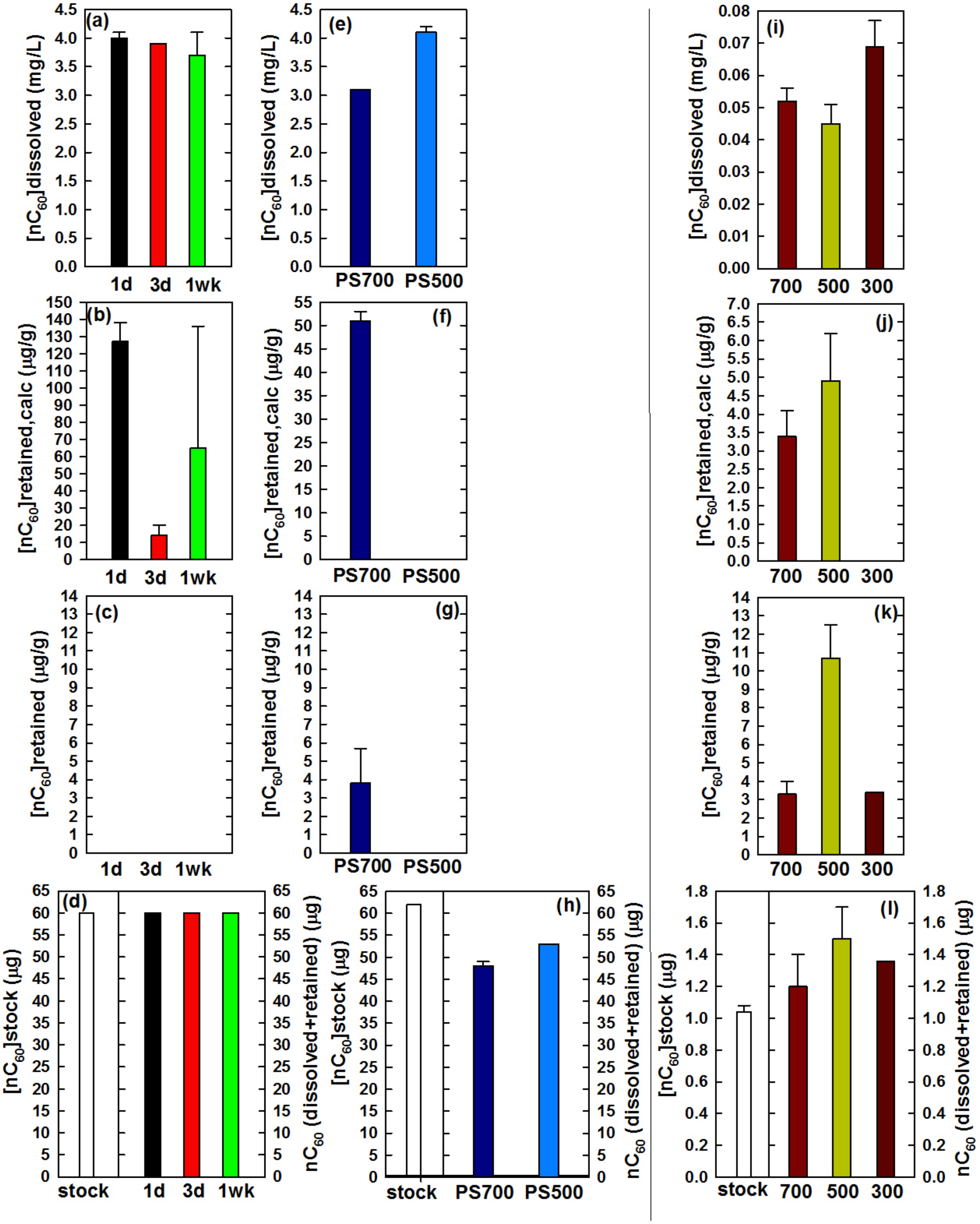

Figure S6. Retention kinetics of nC_{60}-sonicate (a-d) on PS300 (a-d) and 1 d equilibration of nC_{60}-sonicate (e-h) and nC_{60}-stir (i-l) on PS300-700 at 5 (a-d, i-l) and 20 (e-h) g L^{-1} biochar loadings. In d, h, and l, left y-axis is for $[nC_{60}]_{stock}$, and right y-axis is for the mass balance (dissolved+retained). Values in b, f, and j were calculated using Equation 1. All values are given as mean±s.d. of duplicate experiments.

V. Dispersion of 350 and 700 °C pecan shell biochar by nC60-stir

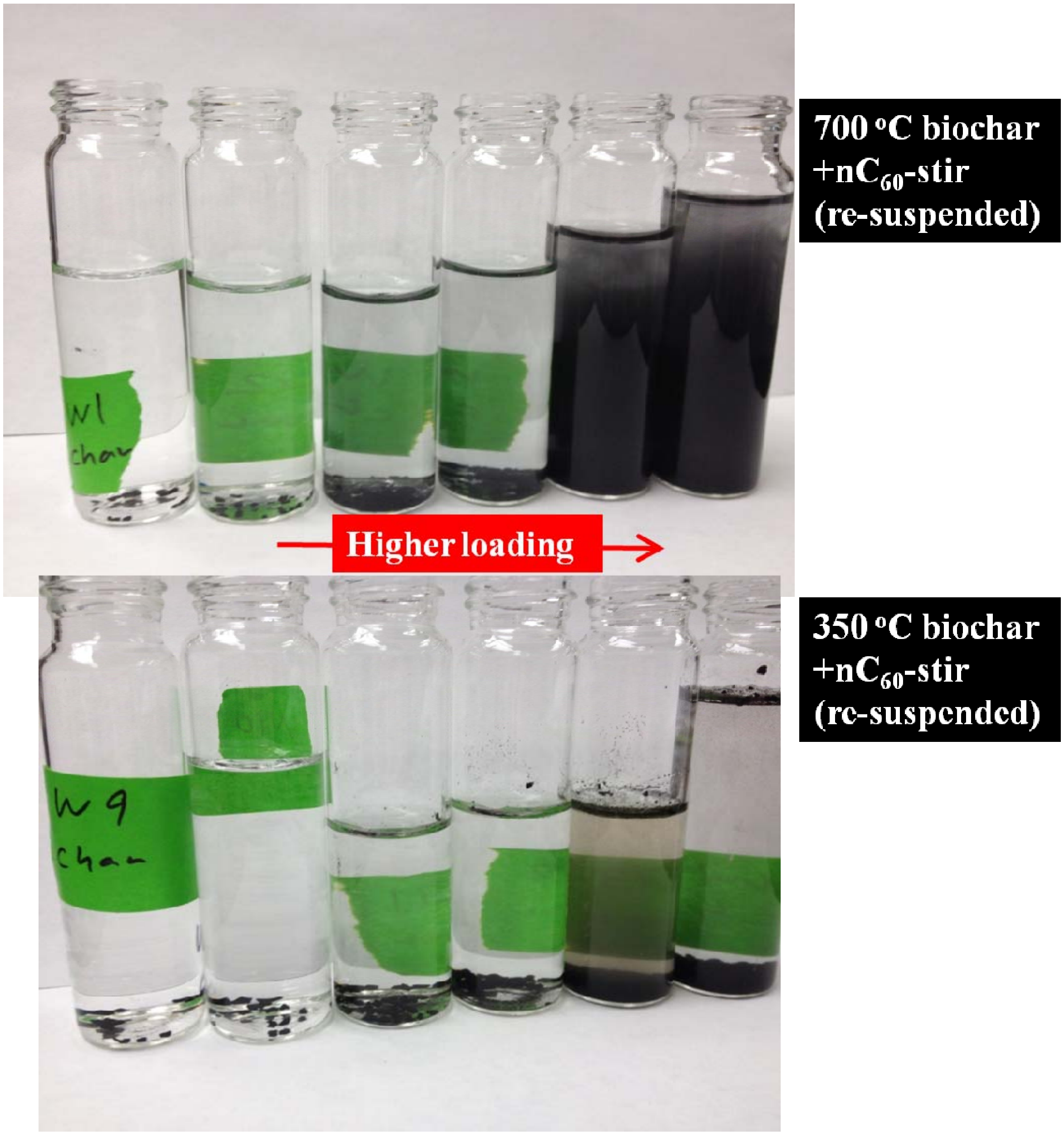

Figure S7. PS700 (top) and PS350 (bottom) after 3d equilibration with nC_{60}-stir, and then resuspended in water by replacing the supernatant with water. The PS700 and PS350 loadings were 1, 4, and 20 g L^{-1} from left to right, each in duplicate.

VI. Reported $[nC_{60}]_{stock}$

Literature survey indicates reproducibility problems in both production[10] and quantification of nC_{60}-stir (summarized in Table S2, Supporting Information). Total organic carbon (TOC) analyzer measured 3.34 mg-C L^{-1} (by combustion of nC_{60}-stir at 680 °C; 11.62 mg-C L^{-1} for nC_{60}-sonicate),[11] 4 mg-C L^{-1},[12] to as low as 0.35 mg-C L^{-1} (3 wk stirring).[13] UV absorbance (355 nm) of toluene extract measured 0.18-0.26 mg L^{-1} for nC_{60} stock solutions stirred in DDW for 2 wk to 11 months with 0-10 mM added NaN_3 or NaCl.[14] In a separate study, UV/visible measurement (336 nm) of repeated (3 extraction cycles) toluene extracts measured 1.2 mg L^{-1} nC_{60}.[15] Concentrations measured by HPLC are typically several-fold lower than UV absorbance or TOC, and ranged from 40 µg L^{-1} after 8 d stirring,[16] 126-244 µg L^{-1} after 99-1,075 d stirring[17] (70:30 toluene-methanol extraction with NaCl), to as low as 7.2 µg L^{-1}.[18] Variations in the size and concentration of nC_{60}-stir stock solutions (Figures 1 and Table S2) and disappearance upon filtration (thin lines in Figure 1) motivated us to rigorously characterize the structure of nC_{60}-stir (Figure 2).

Table S2. Reported $[nC_{60}]_{stock}$.

Stock	Analytical method	$[nC_{60}]$	Ref
nC_{60}-stir (40 d)	TOC by combustion	3.34 mg-C L^{-1}	Chen&Elimelech (2009)
nC_{60}-sonicate	TOC by combustion	11.62 mg-C L^{-1}	Chen&Elimelech (2009)
nC_{60}-stir (40 d)	TOC by combustion	4 mg-C L^{-1}	Xiao et al. (2011)
nC_{60}-stir (3 wk)	TOC by combustion	0.35 mg-C L^{-1}	Li et al. (2009)
nC_{60}-stir (3 wk)	UV/vis (355 nm)	0.18-0.26 mg L^{-1}	Dhawan (2009)
nC_{60}-stir (2 wk)	UV/vis (336 nm)[a]	1.2 mg L^{-1}	Chang et al. (2012)
nC_{60}-stir (2 wk)	HPLC	40 µg L^{-1}	Issacton&Bouchard (2010)
nC_{60}-stir (99-1750 d)	HPLC	126-244 µg L^{-1}	Issacton et al. (2011)
nC_{60}-stir (192 h)	HPLC	7.2 µg L^{-1}	Terashima&Nagao (2007)

[a]Sum of triplicate extraction.

VII. nC_{70}-sonicate characterization

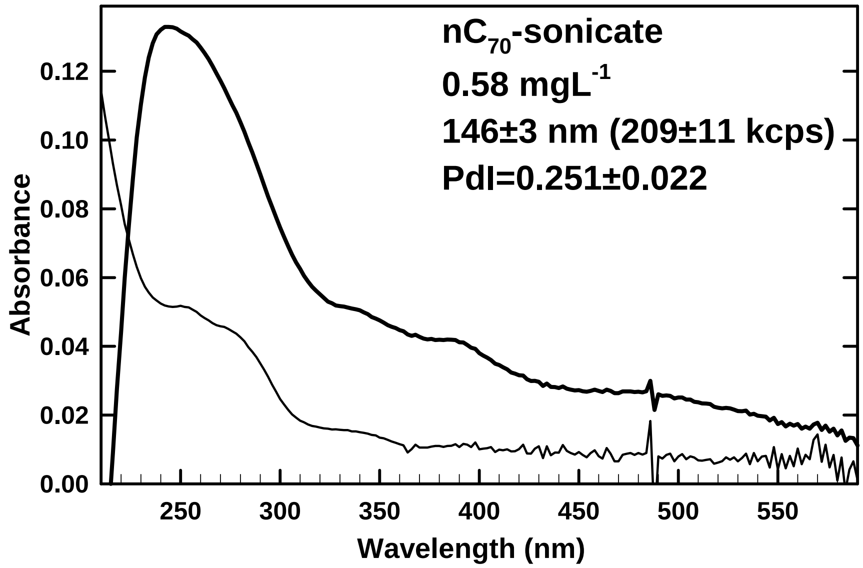

Figure S8. Concentration (determined by UV-HPLC), size, polydispersity index, and count rate (from triplicate DLS analyses), and UV/visible spectra (thin lines were obtained immediately after PTFE syringe filtration) of aqueous nC_{70} stock solution produced by sonication in this study.

VIII. Interpretation of Figure 2c

In Figure 2, the pore diameter is measured from the intensity profile across the pore. Figure 2c shows the intensity of the various pixels across the pore. The intensity is higher for the brighter pixels and lower for the darker pixels. We assumed the pore diameter to be the distance between the two pixels with the highest intensity across the darker region. However, as we are measuring from the center of one pixel to the center of another pixel, we have an error of 0.02 nm, which is equal to the pixel size.

References

(1) ASTM D7582 *Standard Test Methods for Proximate Analysis of Coal and Coke by Macro Thermogravimetric Analysis*; American Society for Testing and Materials, West Conshohocken, PA (2010).

(2) Boehm, H. P., Chemical identification of surface groups. *Advan. Catal.* **1966,** *16*, 179-274.

(3) Uchimiya, M.; Wartelle, L. H.; Klasson, K. T.; Fortier, C. A.; Lima, I. M., Influence of pyrolysis temperature on biochar property and function as a heavy metal sorbent in soil. *J. Agr. Food Chem.* **2011,** *59*, 2501–2510.

(4) Shinogi, Y.; Kanri, Y., Pyrolysis of plant, animal and human waste: Physical and chemical characterization of the pyrolytic products *Bioresour. Technol.* **2003,** *90*, 241-247.

(5) Lattao, C.; Cao, X.; Mao, J.; Schmidt-Rohr, K.; Pignatello, J. J., Influence of molecular structure and adsorbent properties on sorption of organic compounds to a temperature series of wood chars. *Environ. Sci. Technol.* **2014,** *48*, 4790-4798.

(6) Braida, W. J.; Pignatello, J. J.; Lu, Y. F.; Ravikovitch, P. I.; Neimark, A. V.; Xing, B. S., Sorption hysteresis of benzene in charcoal particles. *Environ. Sci. Technol.* **2003,** *37*, 409-417.

(7) Xiao, F.; Pignatello, J. J., Interactions of triazine herbicides with biochar: Steric and electronic effects. *Water Res.* **2015,** *80*, 179-188.

(8) Bouchard, D.; Ma, X.; Isaacson, C., Colloidal properties of aqueous fullerenes: Isoelectric points and aggregation kinetics of C60 and C60 derivatives. *Environ. Sci. Technol.* **2009,** *43*, 6597-6603.

(9) Uchimiya, M., Influence of pH, ionic strength, and multidentate ligand on the interaction of Cd^{II} with biochars. *ACS Sustainable Chem. Eng.* **2014,** *2*, 2019-2027.

(10) Chang, X.; Vikesland, P. J., Uncontrolled variability in the extinction spectra of C_{60} nanoparticle suspensions. *Langmuir* **2013,** *29*, 9685-9693.

(11) Chen, K. L.; Elimelech, M., Relating colloidal stability of fullerene (C_{60}) nanoparticles to nanoparticle charge and electrokinetic properties. *Environ. Sci. Technol.* **2009,** *43*, 7270-7276.

(12) Xiao, Y.; Chae, S. R.; Wiesner, M. R., Quantification of fullerene (C_{60}) in aqueous samples and use of C_{70} as surrogate standard. *Chem. Eng. J.* **2011,** *170*, 555-561.

(13) Li, Q.; Xie, B.; Yu, S. H.; Xu, Y., Kinetics of C_{60} fullerene dispersion in water enhanced by natural organic matter and sunlight. *Environ. Sci. Technol.* **2009,** *43*, 3574-3579.

(14) Dhawan, A.; Taurozzi, J. S.; Pandey, A. K.; Shan, W.; Miller, S. M.; Hashsham, S. A.; Tarabara, V. V., Stable colloidal dispersions of C_{60} fullerenes in water: Evidence for genotoxicity. *Environ. Sci. Technol.* **2006,** *40*, 7394-7401.

(15) Chang, X.; Duncan, L. K.; Jinschek, J.; Vikesland, P. J., Alteration of nC_{60} in the presence of environmentally relevant carboxylates. *Langmuir* **2012,** *28*, 7622-7630.

(16) Isaacson, C. W.; Bouchard, D. C., Effects of humic acid and sunlight on the generation and aggregation state of aqu/C_{60} nanoparticles. *Environ. Sci. Technol.* **2010,** *44*, 8971-8976.

(17) Isaacson, C.; Zhang, W.; Powell, T.; Ma, X.; Bouchard, D., Temporal changes in aqu/C_{60} physical-chemical, deposition, and transport characteristics in aqueous systems. *Environ. Sci. Technol.* **2011,** *45*, 5170-5177.

(18) Terashima, M.; Nagao, S., Solubilization of [60]fullerene in water by aquatic humic substances. *Chem. Lett.* **2007,** *36*, 302-303.
